# The Impact of Inflation and Its Uncertainty on Pharmaceutical Prices: Evidence from Iran

**DOI:** 10.22037/ijpr.2020.114071.14646

**Published:** 2021

**Authors:** Maryam Soleimani Movahed, Aziz Rezapour, Sajad Vahedi, Hassan Abolghasem Gorji, Rafat Bagherzadeh, Ali Nemati, Gholamreza Nemati, Saeed Mohammad-pour

**Affiliations:** a *Department of Health Economics, School of Health Management and Information Sciences, Iran University Of Medical Sciences, Tehran, Iran. *; b *Health Management and Economics Research center, Iran University of Medical Sciences, Tehran, Iran. *; c *Department of Health Care Management, School of Health, Ahvaz University of Medical Sciences, Ahvaz, Iran. *; d *Department of Health Services Management, School of Health Management and Information Sciences, Iran University of Medical Sciences, Tehran, Iran. *; e *Department of English Language, School of Health Management and Information Sciences, Iran University of Medical Sciences, Tehran, Iran. *; f *Department of Economics, School of Administrative and Economic Sciences, Lorestan University, Khorramabad, Iran.*

**Keywords:** Inflation Uncertainty, Inflation, Pharmaceutical Prices, EGARCH

## Abstract

Pharmaceutical productions are recognized as an essential commodity in the economical literature; therefore, an increase in their prices leads to an increase in the household budget. Currently, about 15-20% of the entire health expenditure in Iran is allocated to the pharmaceutical sector. This study aimed to investigate the effect of inflation and its uncertainty on inflation in pharmaceutical prices in Iran. In this study, the monthly time series of consumer price index from 2001 to 2017 was used to calculate inflation uncertainty based on a generalized autoregressive conditional heteroscedasticity model. Hylleberg-Engle-Granger-Yoo test was performed to determine the stationary of the data. Feasibility tests were also used to explore the application of Autoregressive conditional heteroscedasticity family models to these data. The causal relationship between inflation uncertainty and inflation in the pharmaceutical sector was investigated using the Granger causality test. A causal relationship was found between inflation and inflation uncertainty at the 95% confidence interval for the monthly data during the study. It was revealed that Inflation uncertainty did not affect the inflation in the pharmaceutical prices, but inflation can be a cause of pharmaceutical inflation. Although inflation uncertainty has no association with pharmaceutical inflation, it seems that it could affect pharmaceutical inflation through inflation in other sectors. Therefore, adopting appropriate monetary policies aimed at controlling liquidity and inflation can effectively control pharmaceutical prices.

## Introduction

Inflation refers to a continual and gradual increase in prices. The important point in defining inflation is time and a continual increase in prices; that is, prices should continually increase over a time (1). One of the most significant effects of inflation is the uncertainty it creates about future inflation (2-4), meaning that the economic agents are uncertain about the rate and trend of inflationleading to safety savings and investment (5, 6). 

The inflation rate may reflect the economic performance and well-being of an economy (7). It affects both the generalized consumption of market goods and non-market public goods and environmental and health services. (8). In discussing the effects of inflation on health sectors, it is worth noting that pharmaceutical productions are major component of health sectors.

According to the World Bank, pharmaceutical expenditures amount to 20-50 percent of the total health expenditures for developing countries and 12 percent for Organization for Economic Co-operation and Development (OECD) countries (9). The provision of healthy, effective, and good quality medications at a reasonable price is one of the goals in Iran’s 20-year vision plan (10). There are 89 Pharmaceutical companies in Iran (11). The pharmaceutical market in Iran is expected to experience an annual growth of 6.6% in the next 5 years (2017–2022), which would be 0.5% more than the last 5 years and reach $7.857 billion by 2022 (12).

The pharmaceutical market in Iran is completely regulated. Iran Food and Drug Administration (IFDA), as a part of the Iran Ministry of Health and Medical Education, is responsible for controlling all aspects of the pharmaceutical market, including the registration of new medicines (13). The pharmaceutical market in Iran is largely generic. Generic medicines are substitutes for originator medicines with the same quality, safety, and efficacy (14). 

IFDA also controls pharmaceutical prices through pricing commission regulations on a cost-plus basis and comparing selected companies according to published regulations (11). 

The trend of pharmaceutical prices in Iran highlights an increase in pharmaceutical expenditures in recent years. Nevertheless, a comparison between the trend of prices of domestic and imported medicines and the index of commodity prices and consumer price index (CPI) shows an excessive increase in the price of imported medicines(11). Although many prices in the health sector are set based on government interventions and controls, it must be borne in mind that in economic studies, it is common to use medical cost or expenditure data from previous years to project expected medical costs for a current or more recent year. If an analysis requires pooling multiple years of data to achieve sufficient power, a medical price index is appropriate to present estimates as representing a single year (15). Therefore, pharmaceutical price fluctuations can affect health economics forecasts. Considering the important role of pharmaceutical sectors in Iran’s economy and the mutual effect of inflation and inflation uncertainty on pharmaceutical and health sector prices, the current study aimed to investigate the effects of inflation uncertainty, as one of the major signs of macroeconomic instability. 

## Experimental

In this study, monthly data of Consumer Price Index (CPI), Pharmaceutical Price Index (PPI), Health sector Price Index (HPI) (spanning from 2001 to 2017) were collected from the Central Bank of Iran to calculate the inflation in consumer prices index (P_c_), inflation in pharmaceutical prices(P_d_), and inflation in health sector prices (P_h_). Aggregate Health Sector Price indexes are taken from component indexes used for specific health care goods and services. A CPI includes four specific categories of medical care expenditures from the perspective of the consumer’s out-of-pocket price: prescription and nonprescription medicine, medical equipment and supplies, professional services, and hospital and related services (15). In this study, prescription and nonprescription medicines were used as representatives of the pharmaceutical price index.

Due to the balanced access to three variables, *i.e*., CPI, HPI, and PPI, monthly data spanning from 2001 to 2017 were used in this study. To calculate inflation, the growth rate of the variables was obtained by the first-order difference of their logarithms.

The use of traditional methods in econometrics for time-series studies is based on the assumption that variables are stationary. A variable is stationary if its mean, variance and covariance do not change over time; however, when a variable is non-stationary, false regression in time series modeling may be encountered. Therefore, in order to examine the stationary and non-stationary variables, Hylleberg-Engle-Granger-Yoo (HEGY) test was used. The test is an extension of a theory by Dickey-Fuller and is used to test seasonal unit roots. In this paper, this test was used for monthly data.

In the next step, the uncertainty in inflation was investigated to determine if the variance of inflation changed over time; consequently, the data generation of inflation was determined through Autoregressive–moving-average (ARMA) (p, q) process firstly. The number of sentences in Autoregressive AR (p) and the number of moving average sentences MA (q) were calculated by using the method proposed by Box-Jenkins (16). Schwartz information criterion was used to select the best model. To examine the existence of heteroscedasticity, the autoregressive conditional heteroscedastic (ARCH) test was used. If the time-series variance fluctuates due to positive and negative shocks over time, it can be considered as an indicator of uncertainty. Thus, we can model the variance of time series error sentences and examine the uncertainties. In this study, the exponential generalized autoregressive conditional heteroscedasticity (EGARCH) model was used. 

The EGARCH model is as follows:

$\ln\left(\delta_{t}^{2}\right)=\alpha_{0}+\sum_{i=1}^{p}\alpha_{i}\left|\frac{\varepsilon_{t-1}}{\delta_{t-i}}\right|$

$+\sum_{j=1}^{p}\beta_{j}\ln\left(\delta_{t-j}^{2}\right)+\sum_{i=1}^{p}\gamma_{t}\left(\frac{\varepsilon_{t-1}}{\delta_{t-i}}\right)$

 (2)

The model provides the possibility of asymmetric effects of past error terms with the conditional error variance, called the GARCH exponential model. One of the problems of the standard GARCH models is that positive coefficients should be ensured; however, in this study, Exponential GARCH (EGARCH) was estimated to determine the possibility of negative coefficients too. Therefore, any fitted amount was considered as inflation uncertainty. After that, a model for the inflation uncertainty series was extracted.

In the next step, the Granger causality test was used to detect the direction of the causal relationship between inflation uncertainty, inflation, inflation in pharmaceutical prices, and inflation in health sector prices. Granger causality automatically considers two equations for estimating X and Y variables for both directions.

$Y_{t}=\alpha_{0}+\alpha_{1}+\ldots +\alpha_{L}Y_{t-L}$

$+\beta_{1}X_{t-1}+\ldots \beta_{q}X_{t-q}+\varepsilon_{t}$

 (3)

$X_{t}=\alpha_{0}+\alpha_{1}X_{t-1}+\ldots +\alpha_{L}X_{t-L}$

$+\beta_{1}Y_{t-1}+\ldots \beta_{q}Y_{t-q}+\varepsilon_{-t}$

 (4)

The tested null hypothesis in the Granger model was that in the first regression, X was not a Granger cause of Y, and in the second regression, Y was not a Granger cause of X, in other words:

$H_{0}=\beta_{1}=\beta_{2}=\ldots \beta_{q}=0$

 (5)

Moreover, Wald statistic was used to test the above hypothesis:

$F=\frac{({\mathrm{RSS}}_{R}-{\mathrm{RSS}}_{U})/q}{{\mathrm{RSS}}_{U}/(T-2q-1)}$

 (6)

where ${\mathrm{RSS}}_{R}$ is the sum of the squares of the residuals from restricted H_0, _and ${\mathrm{RSS}}_{U}$ is the sum of the squares of unrestricted H_0_. T is the number of observations, and q is the length of the interval in the causal variable; in other words, F compares the sum of the residuals with and without the restriction of H_0 _H_1_. Moreover, the Schwarz criterion was used to determine the interval between variables X and Y. 

## Results

**Figure 1:**
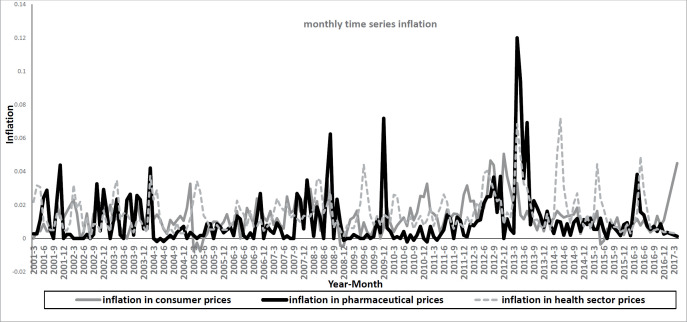
Monthly time series inflation 2001:04 to 2017:03

In this section, the results obtained from the study are presented. As shown in Figure 1, the Consumer Price inflation series had a long-term sinusoidal oscillation and a strong oscillation at short-term, monthly frequency. However, the figure indicates a stable trend in inflation in pharmaceutical and health sector prices in the long-term; furthermore, short-term fluctuations were observed, especially during the implementation of the health sector reform plan.

The HEGY test was used to verify or reject the alternative hypothesis of stationary.

The unit-roots for consumer price inflation, Pharmaceutical price inflation, and health sector price inflation were rejected at the five percent significance level. So these series were stationary. Given that the data generation of inflation was determined through Autoregressive – moving-average, appropriate autoregressive orders and moving average orders will be demonstrated. 

As shown in the table, ARMA (1, 3) was selected based on Schwartz information criteria (Absolute value of the largest number (for data generation process of inflation.

The results of the ARCH heteroscedasticity test demonstrated the existence of the ARCH effect after interval 4, *i.e*., the existence of variance heteroscedasticity, and consequently, uncertainty. 

In the next step, an exponential GARCH model was used to calculate the conditional variance of inflation. The conditional variance series from the model was considered as a substitute for the uncertainty of inflation. The best affirmation from the conditional variance model is expressed in the following equation:

$\ln\left(h_{t}\right)=-2.78808179+0.45419768\left|\frac{\varepsilon_{t-1}}{h_{t-1}^{0.5}}\right|$

$+0.74873904\ln\left(h_{t-1}\right)$

 (7)

After the extraction of the uncertainty series, the casual relationships between inflation uncertainty, inflation, inflation in pharmaceutical prices, and inflation in health sector prices were explored using the Granger causality test. The optimal interval in the estimated Vector autoregression (VAR) equation for examining the causal relationship between inflation and inflation uncertainty, inflation uncertainty and inflation in pharmaceutical prices, as well as inflation uncertainty and inflation in health sector prices, was one. VAR is a stochastic process model used to capture the linear interdependencies among multiple time series. VAR models generalize the univariate autoregressive model (AR model) by allowing for more than one evolving variable.

According to Table 2, there was a causal relationship between inflation and inflation uncertainty at the 99% confidence level for the monthly data during the study, meaning that inflation was the Granger cause of inflation uncertainty, but the reverse causality (from inflation uncertainty to inflation) was not confirmed. Therefore, there was no mutual positive causal relationship between these two variables. There was also no evidence among other variables for the rejection of the null hypothesis and the acceptance of the casual relationship.

Inflation was recognized as the Granger cause of inflation in pharmaceutical prices at the 95% confidence level; however, the reverse was not true. Also, a unilateral Granger causality relationship existed from inflation to inflation uncertainty. Moreover, inflation was the Granger cause of inflation in health sector prices at the 90% confidence level, and as highlighted in Table 2, it was a unilateral relationship. As it can be seen, inflation was the Granger cause of inflation uncertainty and inflation in pharmaceutical prices and health sector prices. Nevertheless, the inflation uncertainty was not the cause of inflation in pharmaceutical prices.

## Discussion

The results of the present study indicated a direct relationship between inflation and inflation uncertainty. However, there was no association between inflation in pharmaceutical prices and inflation uncertainty. This means that pharmaceutical prices were only affected by inflation. 

It should be noted that proper modeling of uncertainty can be effective in the correct prediction of a time series. In this study, the EGARCH model was used to model inflation fluctuations as a representative of inflation uncertainty. One of the important limitations of other methods such as ARCH and GARCH for their symmetry is that they consider the absolute value of the variations in the prediction of the fluctuation and ignore their signs. Thus the effects of negative and positive shocks of the same magnitude on the series fluctuation were equal. In comparison, inflation fluctuations do not respond to the type of news (positive or negative shocks). Therefore, in this study, the asymmetric EGARCH model was used to solve this problem The results indicated asymmetric involvement of negative and positive inflation shocks in the formation of inflation uncertainty. These results were similar to those of the studies conducted by Samimi *et al*. and Entezarkhiz *et* *al*. (17, 18).

According to the findings, Iran has suffered from high inflation and inflation uncertainty in recent decades. Over the last two decades, inflation was a major problem for Iran’s economy. Iran encountered higher inflation with more fluctuations in the 1990s; however, it experienced a downward and at the same time relatively stable trend in inflation trend after conducting anti-inflation policies in 2000. But after five years, the country faced an upward trend in inflation, and since late 2011, the prices have started to abnormally increase with more fluctuations(19). In the context of rising prices, inflation, and subsequent uncertainty, households are more likely to spend their incomes on essential commodities, such as medicine and other health care and less on long-term savings and investment (20). According to the findings of the study by Emamgholi-pour *et al*., the price elasticities of pharmaceutical expenditures for urban and rural households were 0.08 and 0.63, in that order (21). Ravangard conducted a time-series study and found the price elasticity of pharmaceutical expenditures to be less than one(22). Other studies indicate that medicines are necessary commodities for households in Iran (23-25). However, another showed that medicines were non-elastic to the elderly and rich Australians(26). In their study, Shea *et al*. examined the effect of insurance coverage on prescription drugs by Medicare beneficiaries. They found the price elasticity of prescription drugs to be -0.54. Therefore, the rate of change in their consumption was less than the volatility rate of pharmaceutical prices (27). According to the results of this study and the results of the studies mentioned above, medicine, as an essential commodity and inflation uncertainty, does not affect the price of consumption of drugs.

About 96% of all medicines in the pharmaceutical market in Iran, in terms of number/volume, are produced locally, and less than 4% are imported; however, the value of imported medicines is about 40% of the value of the whole market, and most domestic products are made with imported raw material(28). Moreover, the recent sanction by the European Union and the United States in 2012 has caused great concern for the Ministry of Health and Medical Education (MOHME) in Iran to purchase and import medicine and medical equipment to protect people from the effects of these sanctions (28). Due to these sanctions and restrictions, industrial sectors in Iran, including the local pharmaceutical industry, have experienced major problems in buying needed machinery, technology, and even finished products. On the other hand, the national currency (Rial) lost its value against international currencies due to the reduction of international incomes. These problems led to an increase in prices and the pharmaceutical market in Iran(29).

Similarly, the findings of the study by Cheraghali (2013) highlighted the great influence of political sanctions on the pharmaceutical sector, for instance, sanctions on banks and transportation lessen timely access to essential medicines in domestic markets; additionally, the pharmaceutical industry has had serious problems related to importing drugs or raw materials (30). Therefore, it can be concluded that inflation uncertainty can indirectly affect pharmaceutical prices through exchange rate fluctuations and devaluation of the national currency. On the other hand, many price fluctuations in medicines can be controlled under the direct supervision of the government. Direct and indirect subsidies to the pharmaceutical sector could increase government spending on health. Rising government spending will increase inflation despite financial constraints and sanctions. According to the results, rising inflation has increased inflation uncertainty in Iran’s economy. Therefore, government price stabilization policies indirectly affect inflation uncertainty. People also are not aware of the prices of medicines and cannot evaluate their quality (31). This asymmetric information concerning the prices and qualities does exist in other health sectors, but it is much more evident in the pharmaceutical market. It is concluded that the drug market has the behavior of monopoly markets more than other health sectors. Compared to other markets, it is easy to raise prices in a monopoly market; thus, according to the results of the Granger causality test, the increase in drug prices can affect the increase in health sector prices.

Based on the results, the increasing rate of inflation in the health sector was higher than inflation during the study period. The literature confirms the findings (32-34) because health inflation is more complicated than inflation due to many hidden determinants, such as the quality improvement of service delivery, knowledge and experiment enhancement of medical and paramedical staff, and technological development (35). However, the growth of health sector inflation and its determinants differs from healthcare expenditure growth. For example, the GDP growth increased healthcare expenditure but decreased healthcare inflation (33). 

The findings in this report are subject to at least two limitations. First, we had access to limited data, and more recent data on the inflation rate in Iran were not available. Second, there were only a few or no studies conducted on the same topic. These limitations contributed to our limited knowledge about inflation and pharmaceutical prices. Therefore, further studies are needed to explore the relationship between the pharmaceutical inflation rate and other variables, such as foreign exchange rates and urbanization.

**Table 1:** Autoregressive and moving average orders based on Schwartz information criteria

	**0**	**AR (1)**	**AR (3)**	**AR (11)**	**AR (13)**	**AR (25)**
Zero	-6.427557	-6.732714	-6.533730	-6.424443	-6.402196	-6.402575
MA (1)	-6.664835	-6.722538	-6.693693	-6.643295	-6.639164	-6.644807
MA (2)	-6.487923	-6.708724	-6.532833	-6.480800	-6.460452	-6.462676
MA (3)	-6.499203	-6.734362	-6.523685	-6.490906	-6.476730	-6.472731

**Table 2:** The results of the Granger causality test

**Prob.**	**F-Statistic**	**Null Hypothesis**
2.E-07	28.9362	Inflation does not Granger Cause uncertainty
0.6387	0.22123	Uncertainty does not Granger Cause inflation
0.4987	0.45956	Pharmaceutical inflation does not Granger Cause uncertainty
0.3449	0.89675	Uncertainty does not Granger cause pharmaceutical inflation
0.1051	2.65274	Inflation in the health sector does not Granger Cause uncertainty
0.6562	0.19877	Uncertainty does not Granger Cause inflation in the health sector
0.3469	0.88918	Pharmaceutical inflation does not Granger Cause inflation
0.0258	5.04725	Inflation does not Granger Cause pharmaceutical inflation
0.0583	3.63058	Inflation does not reflect Granger inflation in the health sector
0.1177	2.47077	Inflation in the health sector does not Granger Cause inflation
0.1746	1.85671	Inflation in the health sector does not Granger Cause pharmaceutical inflation
0.1998	1.65581	Pharmaceutical inflation does not Granger Cause inflation in the health sector

## Conclusion

In this study, a significant relationship was found between inflation and inflation uncertainty in Iran. Furthermore, inflation affected the inflation in pharmaceutical prices, but inflation uncertainty did not affect inflation in pharmaceutical prices. This means that consumer expectations from and reactions towards inflation may indirectly affect pharmaceutical prices through inflation rather than inflation uncertainty. 

As lower inflation could cause economic stability; however, a rapid policy response to inflation could not only reduce inflation uncertainty but also decrease the inflation in the healthcare and pharmaceutical sectors. Therefore, pharmaceutical policymakers are recommended to support the production of generic and essential medicines to control the rising pharmaceutical prices and reduce the import of pharmaceutical productions. 

Due to the importance of medicine in the health sector, price transparency can be used to reduce price volatility.

## Authors’ contributions

The study has been connived and performed by Saeed Mohamadpour. Other authors contributed to the study equally. Maryam Soleimani Movahed supervised the study. All the authors were involved in substantially reviewing and revising the manuscript. All the authors have read and approved the final draft of the manuscript. 
